# The development and evaluation of a community-based clinical diagnosis tool and treatment regimen for postpartum sepsis in Bangladesh and Pakistan

**DOI:** 10.1186/s12978-016-0124-1

**Published:** 2016-02-25

**Authors:** L. A. Bartlett, A. E. LeFevre, F. Mir, S. Soofi, S. Arif, D. K. Mitra, M. A. Quaiyum, S. Shakoor, M. S. Islam, N. E. Connor, P. J. Winch, M. E. Reller, R. Shah, S. El Arifeen, A. H. Baqui, Z. A. Bhutta, A. Zaidi, S. Saha, S. A. Ahmed, on behalf of the ANISA-Postpartum Sepsis Study Group

**Affiliations:** Department of International Health, Johns Hopkins Bloomberg School of Public Health, Baltimore, Maryland USA; Department of Paediatrics and Child Health, Division of Women & Child Health, The Aga Khan University, Karachi, Pakistan; Centre for Reproductive Health, icddr,b, Dhaka, Bangladesh; Department of Microbiology, The Child Health Research Foundation, Dhaka Shishu Hospital, Dhaka, Bangladesh; Division of Medical Microbiology, Department of Pathology, The Johns Hopkins University School of Medicine, Baltimore, Maryland USA; Centre for Child and Adolescent Health, icddr,b, Dhaka, Bangladesh; Department of Population, Family and Reproductive Health, Johns Hopkins Bloomberg School of Public Health, Baltimore, Maryland USA

**Keywords:** Maternal, Puerperal sepsis, postpartum, Pakistan, Bangladesh, Endometritis, Perinatal, Low-resource countries, Infection, Algorithm, Methods, South Asia

## Abstract

**Background:**

Postpartum sepsis accounts for most maternal deaths between three and seven days postpartum, when most mothers, even those who deliver in facilities, are at home. Case fatality rates for untreated women are very high. Newborns of ill women have substantially higher infection risk.

**Methods/Design:**

The objectives of this study are to: (1) create, field-test and validate a tool for community health workers to improve diagnostic accuracy of suspected puerperal sepsis; (2) measure incidence and identify associated risk factors and; (3) describe etiologic agents responsible and antibacterial susceptibility patterns. This prospective cohort study builds on the Aetiology of Neonatal Infection in South Asia study in three sites: Sylhet, Bangladesh and Karachi and Matiari, Pakistan. Formative research determined local knowledge of symptoms and signs of postpartum sepsis, and a systematic literature review was conducted to design a diagnostic tool for community health workers to use during ten postpartum home visits. Suspected postpartum sepsis cases were referred to study physicians for independent assessment, which permitted validation of the tool. Clinical specimens, including urine, blood, and endometrial material, were collected for etiologic assessment and antibiotic sensitivity. All women with puerperal sepsis were given appropriate antibiotics.

**Discussion:**

This is the first large population-based study to expand community-based surveillance for diagnoses, referral and treatment of newborn sepsis to include maternal postpartum sepsis. Study activities will lead to development and validation of a diagnostic tool for use by community health workers in resource-poor countries. Understanding the epidemiology and microbiology of postpartum sepsis will inform prevention and treatment strategies and improve understanding of linkages between maternal and neonatal infections.

## Background

In 2013, over 30,000 maternal deaths (11 %) were attributed to postpartum sepsis, the third most frequent cause of the approximately 290,000 maternal deaths worldwide [1, 2]. Very nearly all these deaths occurred in low-resource settings. The region with the greatest proportion of maternal deaths due to sepsis was South Asia (14 %) [3]. Postpartum sepsis is highly preventable through interventions that are readily available and relatively low-cost. Historical data demonstrates a pronounced fall in maternal mortality in developed countries in the mid-20th century; a substantial proportion of this decline was attributed to the prevention and appropriate treatment of maternal infections and sepsis [4]. Before antibiotics and etiologic studies, case fatality rates exceeded 20 % [5, 6]. Where appropriate antibiotic treatment is available [7], case fatality rates as low as 2 % are attainable [8].

Sepsis morbidity rates are 20-fold higher than mortality rates, with complications including septicemia, shock, peritonitis, or abscess formation requiring surgery [9, 10]. Long-term consequences, especially with delayed or incomplete treatment, include chronic pelvic inflammatory disease and bilateral tubal occlusion leading to compromised future fertility [11]. Further, there is serious risk for infections transmitted to newborns either vertically during the antepartum period or by direct contact during delivery [9, 10].

The incidence of postpartum (PP) sepsis varies worldwide, with reports between 2–10 % and varies by risk factors which include location of delivery (facility vs. home), low socioeconomic status, poor nutrition, anemia, prolonged labor, premature rupture of membranes, multiple pregnancies, primiparity, being overweight and the type of delivery (caesarean versus vaginal), more than 5 vaginal examinations during labor, other obstetrical maneuvers, no use of antibiotic prophylaxis, and other factors [12, 13]. The World Health Organization (WHO) used an estimate of 5 % incidence for the Global Burden of Diseases (GBOD) work [11].

While there are other causes of serious maternity-related infection among postpartum women (e.g., mastitis), this study focused on PP sepsis caused by endometritis due to its dominant attribution to severe morbidity or death. The WHO defines endometritis as: “The infection of the genital tract occurring at any time between the onset of the rupture of membranes or labour and the 42nd day postpartum in which fever and one or more of the following are present: pelvic pain, abnormal vaginal discharge or odor, and delay in the rate of reduction of size of the uterus” [14]. The standard treatment for PP endometritis is a combination of broad-spectrum intravenous antibiotics [15–17]. However, there are currently no global standard guidelines for oral therapies in resource-poor regions—a lack that impacts women who have limited access to adequate healthcare facilities. Epidemiologic evidence on the timing of onset of conditions suggest that the majority of maternal deaths occur on the day of childbirth due to haemorrhage and hypertensive disorders [18]. However, an estimated 13 % of maternal deaths occur between days 3–7, with endometritis being the most frequent cause of death during this time period [12, 19, 20]. This is opposite the pattern for other direct causes of maternal death including haemorrhage and pregnancy-induced hypertension, where more than 90 % of these deaths occur during the first 48 hours after birth [19]. More recent data report declines in intrapartum deaths by more than 35 %, such that more than a third (36 %) of maternal deaths occur in the time period from 24 hours to 42 days after delivery - more than either the antepartum (25 %), intrapartum and immediate postpartum (<24 hours PP: 28 %), or late PP period (>42 days PP: 12 %) [2]. The timing of PP sepsis, coupled with high rates of home deliveries in many low-resource settings [21] has meant that most sepsis cases and deaths occur at home [12, 20].

Until recently, strategies to prevent maternal sepsis emphasized facility-based interventions: infection prevention, early identification, and treatment. Efforts to reduce maternal mortality due to sepsis, particularly in South Asia, are hampered by the large proportion of births which occur outside of the formal health sector, where only 41 % of births in South Asia are attended by a skilled birth attendant [21]. The most recent Demographic and Health Surveys (DHS) indicate that 37 % of births in Bangladesh (57 % in urban areas and 31 % in rural areas - where almost 80 % of births occur), and 48 % of births in Pakistan (68 % urban and 40 % rural- where 70 % of births occur), respectively take place in health facilities [22–24]. For women who do access facility services, variable quality of obstetric care and limited duration of stay after delivery suggest that facility care alone does not necessarily protect women from developing infections.

Evidence of the effect of community-based interventions to reduce maternal sepsis mortality remains scarce [25, 26], and is limited to interventions to *prevent* maternal sepsis, not detect and treat it. Training traditional birth attendants and supplying them with clean delivery kits has been associated with reduced infection and maternal mortality in Pakistan [27], Egypt [28] and Tanzania [29], but not in Bangladesh [30]. In Pakistan, odds of puerperal sepsis were greatly reduced among the trained community health workers (CHWs) (OR 0.17, 95 % CI 0.13-0.23) compared to the controls and there was a non-significant reduction in maternal mortality (OR 0.74, 95 % CI 0.45-1.23). A recent systematic review also found that clean delivery kits presented as part of a package of interventions that involve training CHWs on recognition and referral and educating women were also associated with reductions in maternal sepsis [31]. A cluster-randomized controlled trial of a participatory intervention among women’s groups in India found increases among intervention clusters in safe delivery practices, including clean birth kit usage and hand washing among birth attendants [32]. Vaginal cleansing (chlorhexidine or other antiseptic) does not appear to prevent maternal or newborn infection, although a non-significant decline in endometritis has been documented (risk ratio 0.83; 95 % confidence interval 0.61 to 1.13) [33]. Evidence also suggests that supplementation with micronutrients can reduce sepsis mortality [34–36]. However, findings from recent community-based randomized trials in Ghana and Bangladesh suggest that supplementation with vitamin A does not significantly reduce maternal mortality (either sepsis-related or all-cause) [37, 38], nor does the WHO recommend routine vitamin A supplementation during pregnancy [39, 40]. New postnatal care guidelines emphasize the risk following discharge from a facility, and includes recommendations to educate women about symptoms of sepsis, and to assess for temperature, lochia and uterine tenderness during postnatal checks.

However, there is virtually no evidence about community-based methods for maternal PP sepsis *diagnoses and treatment.* This study seeks to address that gap. Many of the risk factors for maternal PP sepsis are also those that also put newborns at risk. In recent years, advances made in the development and use of simple clinical diagnostic algorithms and referral or treatment through front-line health workers. These programs have achieved reductions of 34 to 67 % in neonatal mortality [41, 42]. This information has been taken up at national and international program and policy levels – providing an opportunity to reduce newborn mortality in other low-resource settings [43]. The majority of maternal PP sepsis deaths are almost completely avoidable through using aseptic methods, along with timely diagnosis and prompt treatment for those women who develop infection.

**Fig. 1:**
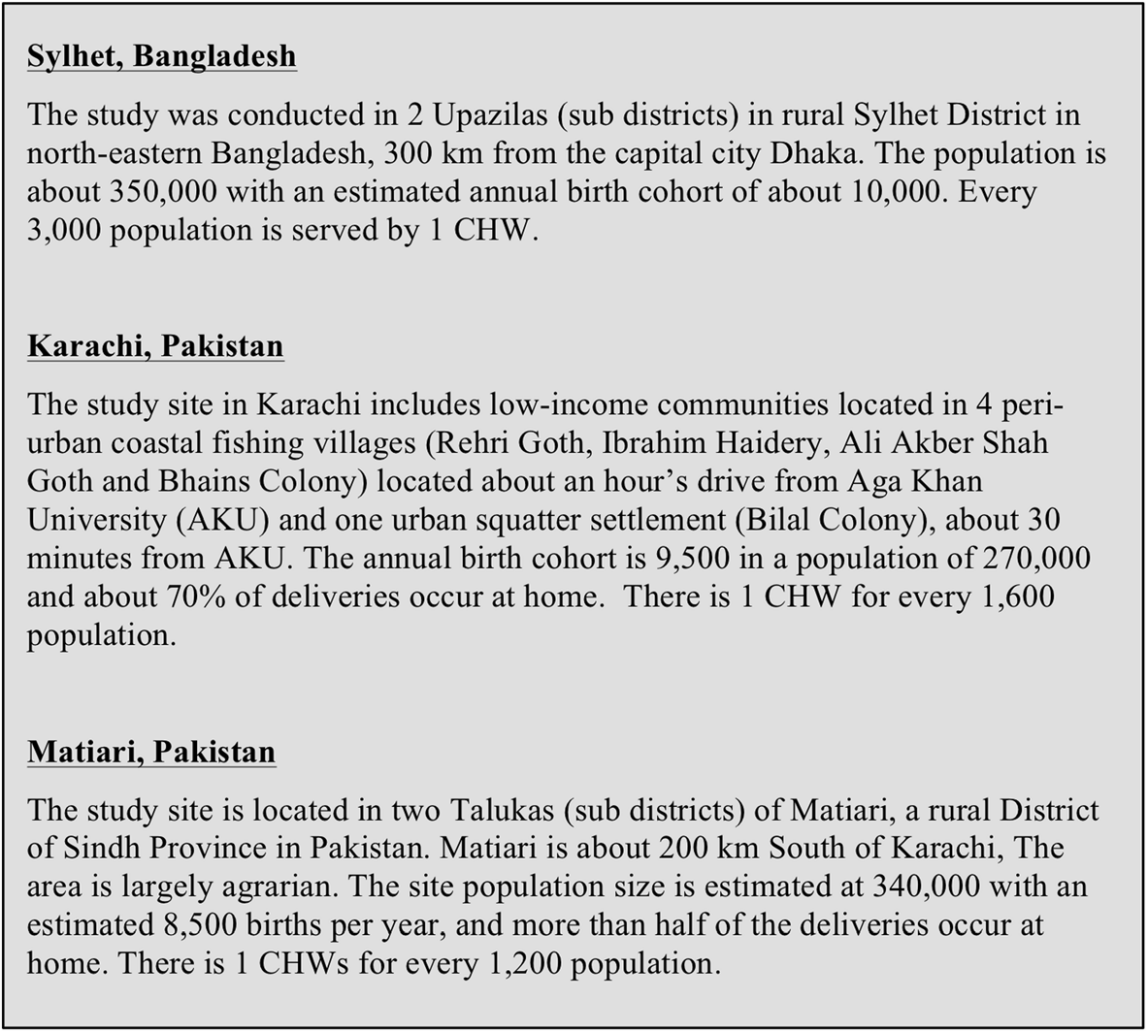
Study Sites

This study, Development of a community-based presumptive clinical diagnosis algorithm and treatment regimen for maternal puerperal sepsis in South Asia is a supplement to the Bill and Melinda Gates Foundation funded Aetiology of Neonatal Infection in South Asia (ANISA) study which was established as a multi-country research project to determine the incidence and etiology of community-acquired neonatal infections in multiple sites through prospective birth surveillance systems. It builds upon previous work to detect and manage newborn sepsis in the community [42, 44]. In this study follow up of postpartum women by CHWs was added to the surveillance system with the overall goal to detect and treat women with PP sepsis and avert sepsis-related maternal deaths. It was implemented in three sites: rural Sylhet, Bangladesh, urban Karachi and rural Matiari, Pakistan (Fig. 1). The objectives of this study are to (1) develop, field test, and validate a locally-adapted algorithm for CHWs to assess both the ability of the CHW to identify the signs and symptoms of PP sepsis in the draft algorithm and the effectiveness of the algorithm as a tool to identify women with PP sepsis; (2) measure PP infection incidence and determine risk factors to inform prevention strategies; (3) to determine etiology and antimicrobial susceptibility patterns to inform appropriate community-based empiric antimicrobial regimens.

## Methods/Design

### Study design and procedures

**Fig. 2:**
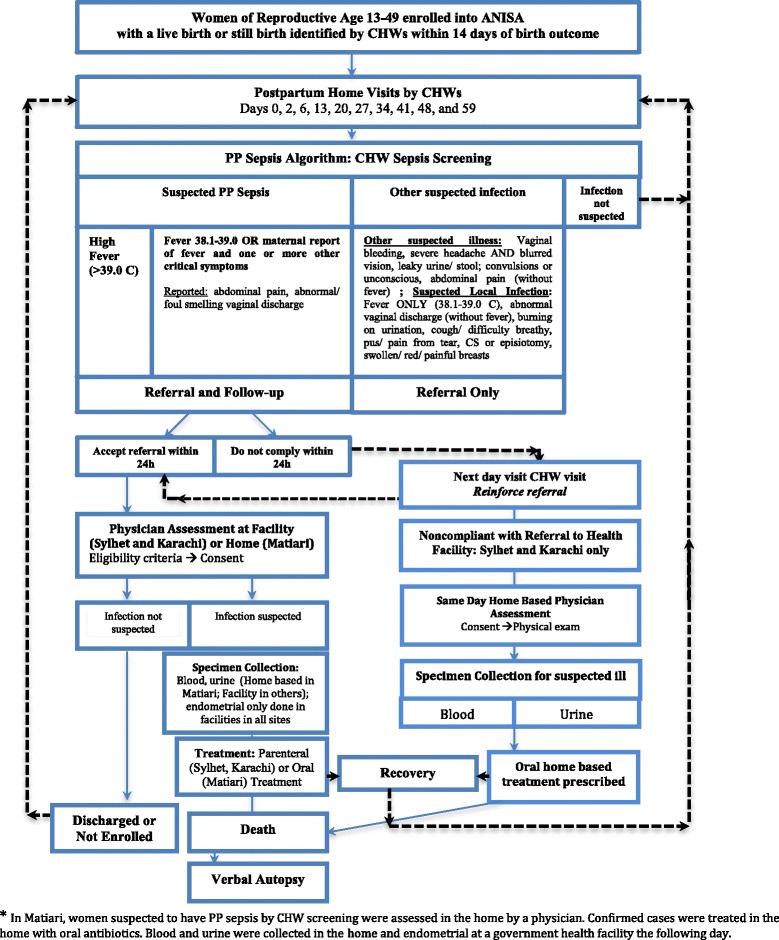
Surveillance system for postpartum maternal infection in Karachi Matiari* and Sylhet

The PP sepsis supplement to ANISA is a partnership between the Johns Hopkins Bloomberg School of Public Health of the Johns Hopkins University, the Child Health Research Foundation and the International Center for Diarrhoeal Disease Research, Dhaka, Bangladesh (icddr,b), Shimantik in Sylhet, Bangladesh, and the Aga Khan University in Karachi, Pakistan. The community-based surveillance systems utilized by ANISA allows CHWs to register married women of reproductive age (13-49 years), identify pregnancies during surveillance home visits conducted every two months, conduct birth preparedness visits at 12–20 weeks and 28–30 weeks of pregnancy (Fig. 2), and carry out 10 postpartum home visits to all enrolled newborns. Details of the ANISA study and site surveillance systems are available elsewhere [45, 46]. The study was implemented in two phases between June 2012 and August 2014: (1) formative research to assist in the development of an algorithm including systematic literature reviews and qualitative research; and (2) algorithm validation and integration into the ANISA newborn surveillance platform which included referral, clinical confirmation, specimen collection and etiologic assessment of PP sepsis episodes by comprehensive testing of clinical samples.

In the formative phase (June to October 2012), in-depth interviews and focus group discussions were carried out among facility and community-based health care providers and beneficiaries to elicit local definitions and understanding of signs and symptoms of PP sepsis. Participant groups included facility-based health providers, women with PP sepsis admitted to facilities, female relatives of women admitted with PP sepsis, community-based healthcare providers for women with PP sepsis before referring them to facilities, recently delivered women in the community, family members of recently delivered women in the community, and community-based unskilled healthcare providers.

Data were transcribed, coded, and analyzed to examine: 1) local terminology corresponding to clinical signs of PP sepsis; 2) care-seeking, sources of care, and treatment given for conditions compatible with PP sepsis in the community. Interim findings were utilized to inform the CHW algorithm content and key issues for implementation, e.g., ensuring a private place for interviews and training CHWs to probe sensitively for additional maternal symptoms and signs. Detailed results are being prepared for publication.

To complement primary data collection, two systematic literature reviews were conducted, implemented jointly and led by WHO and CDC respectively to (1) identify other potential algorithms and possible clinical components; and (2) examine literature on both reported etiology (Requejo J, Widmer M, Bartlett L, Kaur G, Allen T, Gross P, et al. A systematic review of clinical diagnostic algorithms on puerperal sepsis for potential use at the community level. Unpublished) and potential oral antibiotic regimens for women who are unable to undergo facility-based parenteral treatment [47]. These, plus expert consultation, led to a community-based PP sepsis algorithm (Table 1) and supporting procedures for referral, case management, algorithm validation, specimen collection and analysis.

**Table 1:** Simplified algorithm for identifying postpartum maternal infection

Symptoms screened by CHWs	Classification
*High* fever [temperature 102.4 °F (39.1 °C) or higher]	Suspected PP sepsis
Fever [temperature 100.6 °F - 102.3 °F (38.1 °C – 39.0 °C)]	Suspected PP sepsis if fever present at examination or history of fever AND any other sign or symptom listed is present
History of fever	
Lower abdominal or pelvic pain	
Abnormal or foul-smelling discharge	
Severe Vaginal bleeding	Other suspected illness
Severe headache AND blurred vision	
Leaking urine and/or stool	
Convulsions or unconscious	
Lower abdominal pain (without fever)	
Fever only [temperature 100.6 °F - 102.3 °F (38.1 °C – 39.0 °C)]	Suspected Local Infection
History of fever only	
Abnormal or foul-smelling vaginal discharge (without fever)	
Burning upon micturition	
Cough or difficulty breathing	
Pus or pain from tear, c- section or episiotomy wound	
Swollen, red, or painful breast	

The PP sepsis study enrolled consenting women for maternal infection surveillance with live birth and stillbirth outcomes identified within 14 days of the birth outcome. Maternal infection surveillance procedures were integrated into existing ANISA postpartum home visit schedule; on days 0, 2, 6, 13, 20, 27, 34, 41, 48 and 59 postpartum. Presumptive cases of PP sepsis were defined based on measured temperature, maternal history of fever, lower abdominal or pelvic pain, and abnormal or foul-smelling discharge. Women with high fever (>39.0 °C) at the time of assessment were classified as having suspected PP sepsis and referred. Women with either fever (38.0-39.0 °C) or history of fever paired with at least one additional symptom of sepsis (lower abdominal pain, pelvic pain, or abnormal/foul-smelling discharge) are also presumptively classified as having PP sepsis and referred (Table 1). Women with any serious life-threatening health conditions, whether PP sepsis or not, were also referred to tertiary care facilities.

Among suspected cases of PP sepsis, facility (Sylhet, Karachi) and/or home-based (Matiari) physician assessments were carried out. In Sylhet and Karachi, home-based follow up by physicians occurred only among individuals who could not adhere to CHW-recommended referral. Because of the distance between tertiary care facilities and the remote field site in Matiari, study physicians visited CHW-determined suspected PP sepsis cases in their homes. In all instances, physician-confirmed cases were asked to provide biospecimens (urine and blood in the home; endometrial in health facilities) and were prescribed an oral antibiotic treatment regimen. Women who consented to referral and admission to hospital for treatment were managed according to the local standard of care, although adherence to the WHO recommended treatment in the Managing Complications in Pregnancy and Childbirth (MCPC) [48] was encouraged. In the event of death, family members were interviewed to determine likely cause of death under the auspices of another study in the region with the Alliance for Maternal and Newborn Health Improvement (AMANHI).

### Sample size calculation and sampling strategy

A pregnancy cohort of 26,200 women across the three sites over a one-year implementation period was expected, and was rounded up to 28,000 women to account for any change in population size by immigration or birth rates resulting in more pregnancies. We determined that this sample was adequate for measuring outcomes in all three objectives. Using a conservative estimate of 5 % prevalence of PP sepsis among postpartum women, we estimated that ~36 clinical sepsis cases per month on average for each site would be identified; resulting in a total of 1,310 cases per year (Table 2). This sample size is adequate to estimate PP sepsis incidence rate with at least 1 % margin-of-error at α = 0.05. To validate the algorithm for CHWs and its application, study physicians, blinded to the CHW clinical diagnoses, assessed a random sample of suspected PP sepsis (*n* = 75) and healthy women (*n* = 225) to meet the required sample size for assessing sensitivity of 95 % and specificity of 97.5 % with 5 % and 2.5 % margins of errors, respectively, and 5 % Type-I (α) error. Verification bias due to oversampling of control women will be corrected with statistical methods during analysis [49, 50]. The study physicians’ diagnostic decision-making was the gold standard and was standardized using training materials about PP sepsis based on WHO Manual of Complications in Pregnancy and Childbirth diagnostic criteria (fever, chills, lower abdominal pain, purulent, foul-smelling lochia, tender uterus, +/**-** light vaginal bleeding and signs and symptoms of shock). When possible, physician validation was performed in health facilities.

To determine the etiology and antibiotic resistance pattern of PP sepsis bacterial isolates at the three study sites, we anticipated identifying and obtaining consent from 70 % of the estimated 1,310 women with PP sepsis in the birth cohort across the three sites (Table 2). This will allow for robust data on etiology, etiology specific incidence of PP sepsis, and will provide specific antibiotic susceptibility data for detected pathogens. In all instances of physician-confirmed PP sepsis, consent was sought from women who adhered to referral for urine, blood, endometrial and high vaginal swab (HVS) samples (HVS only done in Pakistan). Informed consent was obtained from women with physician-confirmed sepsis with the option for them to consent to provide one, two, all, or none of the specimens (urine, blood and endometrial sample) requested. Endometrial specimens were only collected in hospitals by physicians who received clinical training on the Tao Brush by Cook Medical before use [51].

Surveillance continued until 150 women in each site consented to all three specimens. Among women who declined referral and received a home-based assessment by a study physician, consent was obtained only for home-based collection of urine and blood.

**Table 2:** Study population and expected sepsis cases

Characteristics	Sylhet	Matiari	Karachi	Total
Population	~340000	~340000	~270000	950000
Yearly birth cohort	10200	8500	7500	26200
Conservative estimate of PP sepsis cases (5 % prevalence)	510	425	375	1310

### Laboratory methods

Laboratory testing used standardized cross-site protocols [52]. Site-specific analyses yielded a revised list of pathogens (Table 3) and their relative proportions among women with suspected PP sepsis were compared. A list of recommended antibiotic therapies were postulated after evaluating the results of susceptibility testing. Further details on laboratory methods, diagnostic testing, and quality assurance procedures used in this study are described in Shakoor et al.’s Diagnostic methods to determine microbiology of postpartum endometritis in South Asia: laboratory methods protocol used in the Postpartum Sepsis Study, a prospective cohort study, also published in this journal issue [53].

**Table 3:** List of suspected pathogens

Facultative aerobic bacterial pathogens	Anaerobic bacteria	Atypical bacteria by Real time PCR
(a) Gram-positive	Peptococcus sp.	Chlamydia trachomatis
Beta-hemolytic streptococci—Groups A, B, C, D, F	Peptostreptococcus sp.	Mycoplasma hominis, genitalium
Other streptococci (intermedius, sanguis, etc)	Bacteroides fragilis, bivis, disiens	Uroplasma urealyticum
Enterococci	Clostridia ramosum,	
Staphylococcusaureus	Perfringens, welchii, sordellei	
Aerococcus urinae	Fusobacterium	
(b) Gram-negative		
Gardenerella vaginalis		
Enterobacteriaceae, such as klebsiella pneumoniae, enterobacter, escherichia coli, citrobacter, proteus mirabilis		
Pseudomonas aeruginosa		
Neisseria gonorrheae		

### Analysis plan

The diagnostic performance of the algorithm applied by CHWs in a field setting depends upon the effectiveness of the algorithm as a tool to identify women with PP sepsis and the ability of the CHWs to identify the signs and symptoms of PP sepsis described in the algorithm. Validity measures will be calculated using the gold standard physician assessment. Sensitivity and specificity with 95 % confidence intervals (CI) will be calculated at two levels: identification of individual signs and symptoms and classification of suspected PP sepsis. Agreement between CHW and physician assessments will be evaluated using the Kappa statistic. Change in inter-rater agreement over time will also be calculated to assess any improvement in the ability of the CHWs to accurately clinically diagnose patients.

Population-level incidence of PP sepsis will be calculated based on both CHW and physician determination of whether women have suspected PP sepsis. Misclassification of diagnosis (false positive, false negative) could bias the estimate. This concern will be alleviated by using sensitivity and specificity estimates from the validation results and correct incidence estimates for unbiased results. Multivariate regression will be used to identify risk factor data on demographic, socioeconomic, health status and care-seeking characteristics of pregnant women in the study collected at the time of enrollment and during CHW scheduled prenatal home visits.

### Ethical considerations

Ethics, consent and permissions: A full review for human subject research was conducted by the Institutional Review Board at Johns Hopkins University, by the Ethical Review Committees at the International Center for Diarrhoeal Disease Research, Dhaka, Bangladesh, the Aga Khan University in Pakistan, and the Bangladesh Institute for Child Health for the Child Health Research Foundation in Bangladesh. Informed oral consent was taken at each level as described in detail.

## Discussion

CHWs and mothers expressed their approval that the mothers were also included in the birth surveillance outcomes research in a systematic way. Due to their familiarity with the newborn sepsis algorithm, the CHWs were readily able to understand, adopt and implement the maternal PP sepsis diagnostic and referral algorithm. Because of the intimate nature of an endometrial sample, the consent form was designed so that women could choose to consent to any, all or none of the specimens in order to provide a setting where women may feel comfortable providing less invasive blood and urine, but able to opt out of the endometrial sample. This did not interfere with attaining the sample size of linked specimens.

For women with suspected PP sepsis who could not comply with referral or facility-based treatment, physicians went to their homes to perform the clinical assessment and encourage facility admission for treatment. For those who could not comply, oral antibiotic treatment was prescribed– the regimen was based on the three oral/parenteral antibiotic regimens identified in the literature review, and differed depending on availability and cost in each site [47]. Although there is no global standard for oral treatment, we reasoned that even if considered sub-optimal to parenteral treatment, oral treatment would be more beneficial to women with suspected sepsis than offering no treatment. While our study focused on diagnoses of PP sepsis, women with health complaints for themselves or their newborns were also referred to the nearest facility, with facilitation by the CHWs when necessary.

The study implementation encountered several operational challenges in the beginning that were successfully addressed those with concerted team efforts. In Sylhet site, recruitment of female physicians at the rural sub-district hospitals was a great challenge for the team. Local cultural practices prohibited the use of male physicians in examining postpartum women for PP sepsis and for collection of the endometrial specimens. In collaboration with local community leaders and key stakeholders in Sylhet, two female physicians were recruited and employed by the study. Training and standardization of physicians and CHWs was also a challenge. However, in collaboration with the Gynecology Department of the Sylhet Osmani Medical College (the only teaching hospital in the area), study physicians and CHWs were successfully trained to conduct clinical examinations for PP sepsis.

In all three sites, physicians received additional training to collect endometrial specimens in tertiary facilities under direct supervision of obstetric consultants. Once collected, laboratory analyses were carried out in Sylhet, Bangladesh and Karachi, Pakistan. In the former, anaerobic culture of specimens was a challenge, mitigated in part by the training of senior laboratory staff at Johns Hopkins Hospital in Baltimore, MD USA.

This study will provide an efficient diagnostic tool for community health workers to identify women with suspected sepsis for referral and management in addition to a critical evidence base for understanding the epidemiology and etiology of PP sepsis among women in Bangladesh and Pakistan. Although evidence exists on the feasibility, acceptability and effectiveness of community-based approaches for identification and management of newborn infections through CHWs, this is the first study of its kind to place equal emphasis on maternal puerperal infections and explore options for integration of the two in community settings. New data generated on the epidemiology of PP sepsis at the community level in three South Asian sites may inform future public health action to prevent or manage this life-threatening illness.  If effective, this diagnostic tool can be applied throughout South Asia and other settings where community-based postnatal care could be implemented - contributing to prevention of maternal mortality from PP sepsis.

## Abbreviations

PP: postpartum
CHW: community health worker
HVS: high vaginal swab
ANISA: Aetiology of Newborn Infection in South Asia
MCPC: Managing Complications in Pregnancy and Childbirth
AMANHI: Alliance for Maternal and Newborn Health Improvement
WHO: World Health Organization
GBOD: Global Burden of Disease
DHS: Demographic and Health Surveys
CDC: Centers for Disease Control and Prevention
